# Zirconium-based metal–organic framework gels for selective luminescence sensing†

**DOI:** 10.1039/d0ra09035b

**Published:** 2020-12-21

**Authors:** Shujian Sun, Caifeng Wei, Yali Xiao, Guangqin Li, Jianyong Zhang

**Affiliations:** MOE Laboratory of Polymeric Composite and Functional Materials, School of Materials Science and Engineering, School of Chemical Engineering and Technology, School of Chemistry, Sun Yat-Sen University Guangzhou 510275 China zhjyong@mail.sysu.edu.cn

## Abstract

Metal–organic gelation represents a promising approach to fabricate functional nanomaterials. Herein a series of Zr-carboxylate gels are synthesized from rigid pyrene, porphyrin and tetraphenyl ethylene-derived tetracarboxylate linkers, namely Zr-TBAPy (H_4_TBAPy = 1,3,6,8-tetrakis(4-carboxylphenyl)pyrene), Zr-TCPE (H_4_TCPE = 1,1,2,2-tetra(4-carboxylphenyl)ethylene), and Zr-TCPP (H_4_TCPP = 5,10,15,20-tetrakis(4-carboxyphenyl)porphyrin). The gels are aggregated from metal–organic framework (MOF) nanoparticles. Zr-TBAPy gel consists of NU-901 nanoparticles, and Zr-TCPP gel consists of PCN-224 nanoparticles. The xerogels show high surface areas up to 1203 m^2^ g^−1^. MOF gel films are also anchored on the butterfly wing template to yield Zr-MOF/B composites. Zr-TBAPy and Zr-TCPE gels are luminescent for solution-phase sensing and vapour-phase sensing of volatile organic compounds, and exhibit a significant luminescence quenching effect for electron-deficient analytes. Arising from the high porosity and good dispersion of luminescent MOF gels, rapid and effective vapour-sensing of nitrobenzene and 2-nitrotoluene within 30 s has been achieved *via* Zr-TBAPy film or Zr-TBAPy/B.

## Abstract 2

Zr-based MOF nanomaterials are developed *via* a metal–organic gelation method for rapid and effective luminescence vapour-sensing.

## Graphical abstract

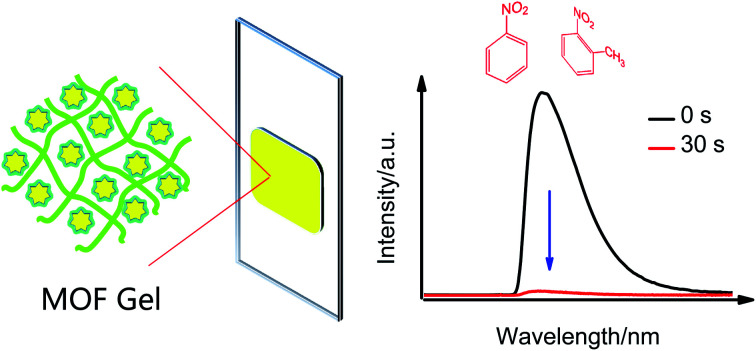

## Introduction

Metal–organic gels (MOGs) have attracted increasing attention in recent years due to their distinctive constituents of metal complexes that enable metal–ligand coordination interaction. Metal–organic gels combine the advantages of stimuli-response of physical gels (weak non-covalent interactions) and stability of chemical gels (strong chemical bonds).^1–4^ Metal–organic gels have found applications in various fields such as sensing,^5–10^ catalysis,^11–14^ environmental remediation,^15,16^ renewable energy conversion,^17–19^ and templates for nanomaterials.^20^ Metal–organic framework (MOF)-based gels are particularly interesting because they incorporate MOF nanoparticles with well-defined microporosity in their gel matrix. MOF gels are assembled in two steps (a) MOF nuclei assembling to form MOF nanoparticles through coordination between metal and organic linker; (b) MOF nanoparticles self-aggregating to form hierarchically porous gel network through disordered coordination perturbation.^21–23^ However, the gelation behaviours of such nanoscale MOF particles remained largely unexplored. Under mild conditions the crystal growth of MOF particles can be surpassed by gelation.^24,25^ In MOGs the process of MOF growth can be effectively controlled by the coordination equilibria compared with traditional solvothermal synthetic route. The resulting MOF nanoparticles are confined in the gel network *via* supramolecular interactions (*e.g.*, hydrogen bonding, π–π stacking). More importantly, the epitaxies of the nanocrystals are inhibited when the coordination is perturbed by other competing interactions during the gelation process, yielding homogeneous and small crystalline particles.

A series of zirconium-based metal–organic gels have been studied, which consist of Zr-MOF nanoparticles, including UiO-66, NH_2_-UiO-66, UiO-67, MOF-801 and MOF-808, *etc.*^26–33^ Through the MOG route, the synthesis of Zr-MOF nanoparticles is simple and rapid under mild conditions.^33,34^ For example, due to the presence of MOF nanoparticles in the gel matrix, the improved performance for adsorption and conversion of CO_2_ was observed for UiO-66-NH_2_ gel.^27^ The MOF route has also been used to *in situ* synthesize UiO-67 nanoparticles on the surface of a bio-photonic crystal template for improved H_2_ production.^32^ These examples have revealed the potential of MOGs as a powerful platform to develop functional MOF nanomaterials.

Zr-MOFs have been applied as chemical sensors because of their high porosity, water stability and chemical stability under sensing conditions.^35–40^ However, crystalline powders of Zr-MOFs are typically used in the sensing. For device fabrication and practical use nanomaterials are highly preferred for fast sensing. MOGs show advantages both in nanomaterial preparation and ready processability. Based on these considerations we develop a series of Zr-tetracarboxylate gels consisting of Zr-MOF nanoparticles assembled from tetratopic carboxylate linkers. The gels are employed to deposit the Zr-MOF nanoparticles on a quartz slide, and on the surface of bio-photonic crystal of butterfly wings as well to get film devices. The devices are tested for solution-phase sensing and vapour-phase sensing of volatile organic compounds.

## Results and discussion

**Fig. 1:**
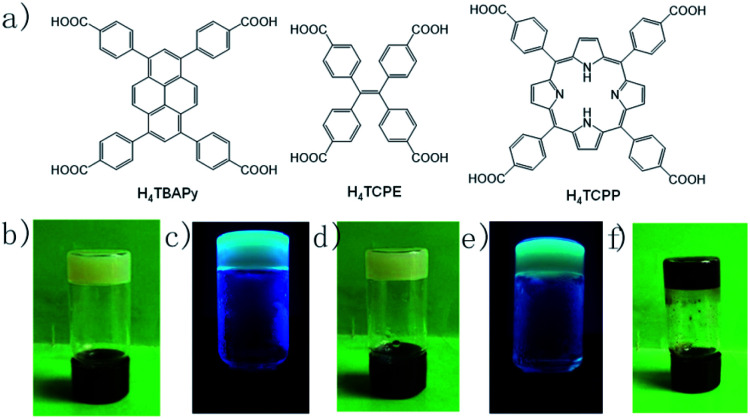
(a) Chemical structures of bridging carboxylic acids, photographic images of (b) Zr-TBAPy gel, (c) Zr-TBAPy gel under 365 nm UV lamp, (d) Zr-TCPE, (e) Zr-TCPE gel under 365 nm UV lamp, and (f) Zr-TCPP gel.

Three tetratopic carboxylic acids were chosen for the gelation (Fig. 1). Zr-TBAPy gel was prepared from the reaction of ZrOCl_2_·8H_2_O and 1,3,6,8-tetrakis(*p*-benzoic acid)pyrene (H_4_TBAPy) in DMF in the presence of HCl (37 wt%) and benzoic acid. Benzoic acid was used as modulator to affect the gelation. A yellow gel was obtained after the mixture was placed in an ultrasound bath for 20 min, and heated in a capped vial at 100 °C for 24 h. Zr-TCPE and Zr-TCPP gels were prepared from tetracarboxyphenyl ethylene (H_4_TCPE) and 5,10,15,20-tetrakis(4-carboxyphenyl)porphyrin (H_4_TCPP), respectively, in a similar way upon heating at 100 °C for 24 h. The wet gels were washed with DMF and EtOH sequentially, and the corresponding xerogels were obtained *via* solvent evaporation under ambient conditions.

**Fig. 2:**
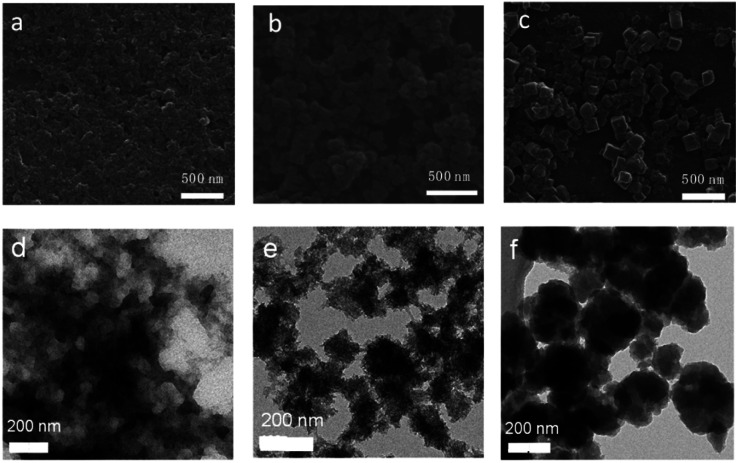
SEM and TEM images of (a and d) Zr-TBAPy, (b and e) Zr-TCPE, (c and f) Zr-TCPP xerogels.

**Fig. 3:**
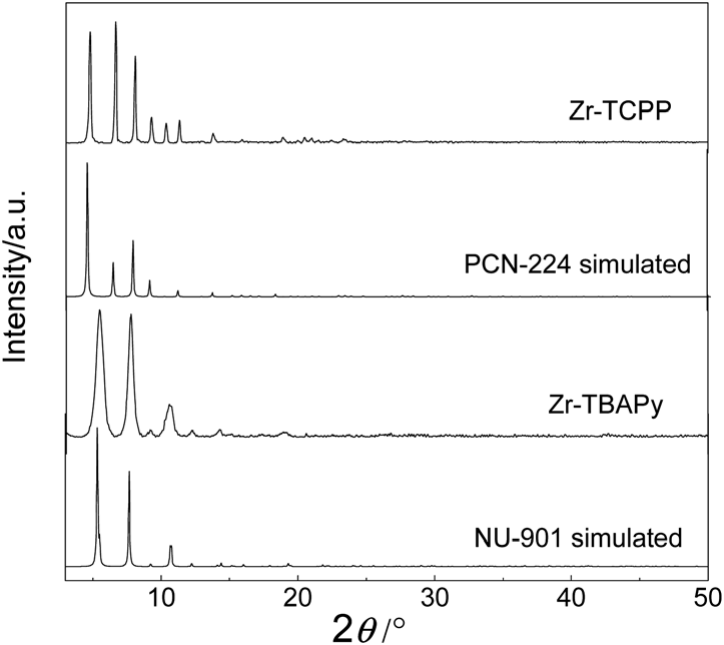
PXRD patterns of Zr-TBAPy, Zr-TCPP xerogels, simulated PCN-224 and NU-901.

SEM and TEM investigations show that Zr-TBAPy has a wormhole-like network with particles size distribution of 75–150 nm (Fig. 2 and S1†). In contrast rodlike microcrystalline materials were yielded with particle sizes in a range of ∼2–10 μm in traditional solvothermal synthesis.^41^ The crystallinity phase of Zr-TBAPy xerogel was examined by powder X-ray diffraction (PXRD) (Fig. 3). The peaks at 5.3° and 7.6° are attributed to the crystal planes (111) and (200) of NU-901.^42–44^ Particularly, Zr-TBAPy gel contained only a single phase of NU-901 with higher yield than that was synthesized previously *via* traditional solvothermal route.^45^ The framework of NU-901 consists of an eight-connected Zr_6_ nodes linked by 1,3,6,8-tetrakis(4-carboxyphenyl)pyrene (TBAPy) ligands with an **scu** network topology and microporous diamond-shaped channels with the pore size of 2.9 nm × 1.2 nm. In fact, mixed phases containing NU-901 and NU-1000 have often been observed in literature.^45^ The present gelation method offers a new route to phase-pure NU-901 under mild conditions while suppressing the formation of NU-1000. According to the Scherrer equation, Zr-TBAPy crystallite nanoparticles were calculated to be *ca.* 6 nm. This is much smaller than the size observed by electron microscopy probably because the aggregation of crystallite nanoparticles leads to large particles.

Zr-TCPE was investigated by SEM and TEM to exhibit regular granular morphology with particle size distribution of 75–120 nm (Fig. 2 and S2†). In contrast related tetraphenylethylene-based coordination polymers were synthesized *via* solvothermal route with octahedron shapes with larger size ∼500 nm.^46^ It indicates that the gelation can restrict the MOF crystal growth to yield nanoparticles. PXRD shows that the Zr-TCPE xerogel is crystalline (Fig. S3†), however, the corresponding structure has not been reported to the best of our knowledge and meanwhile its nanoscale nature prevents further elucidation of the structure.

Zr-TCPP has typical crystallite cubic morphology with 50–150 nm in size as revealed by SEM and TEM (Fig. 2 and S4†). The PXRD pattern was consistent with the PCN-224 phase (Fig. 3).^46–48^ PCN-224 is composed of eight-connected Zr_6_ nodes linked by 5,10,15,20-tetrakis(4-carboxyphenyl)porphyrin (H_4_TCPP) ligands forming a three-dimensional network structure with pore channels of 1.9 nm with a **ftw**-a topology. Each cubic lattice contains eight shared Zr_6_ nodes and six shared porphyrin ligands, and each porphyrin ligand links four Zr_6_ nodes. According to the Scherrer equation, PCN-224 nanoparticles were calculated to be 140 nm.

FT-IR spectra of Zr-TBAPy, Zr-TCPE and Zr-TCPP show intense bands at around 1600 and 1400 cm^−1^, which is associated to the O–C=O asymmetric stretching and symmetric stretching modes of the carboxylate group, respectively (Fig. S5†). The absorptions at around 720 and 650 cm^−1^ are attributed to Zr–O bonds as longitudinal and transverse modes, respectively.^49,50^ N–H bond is located at 3312 cm^−1^ in Zr-TCPP xerogel, which confirms the existence of the uncoordinated nitrogen sites.^46,51^

**Fig. 4:**
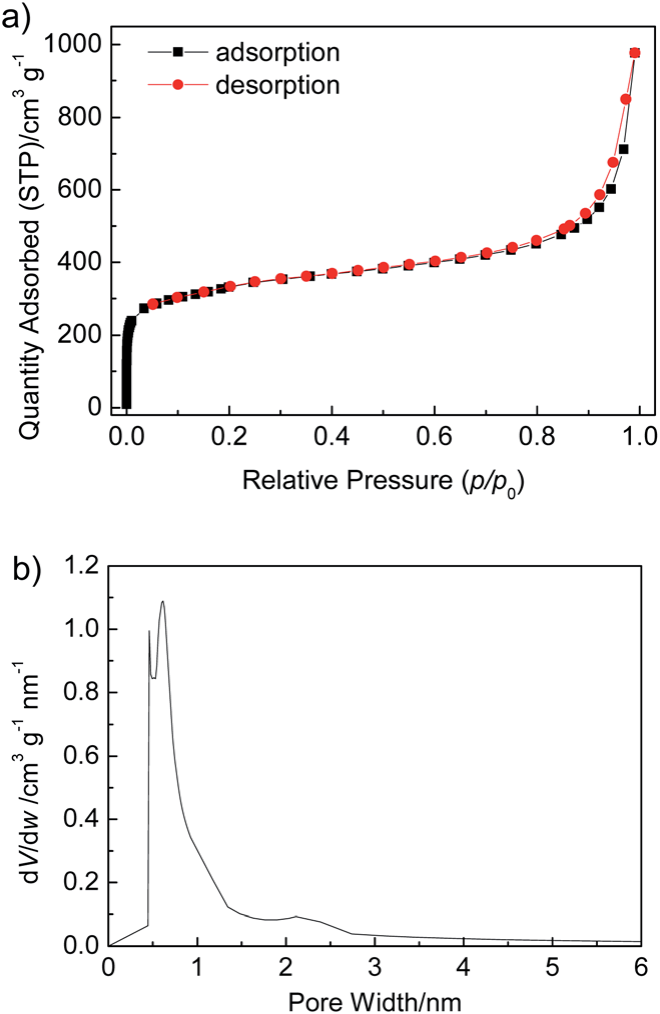
(a) N_2_ adsorption–desorption isotherms measured at 77 K, and (b) Horvath–Kawazoe micropore analysis of Zr-TBAPy xerogel.

To assess the porosity of Zr-TBAPy, Zr-TCPE and Zr-TCPP xerogels, nitrogen adsorption–desorption isotherms were measured at 77 K (Fig. 4, S6 and S7†). The nitrogen isotherms exhibit the combined features of type I and type II isotherm curves according to the IUPAC classification. Obvious nitrogen uptake in the low pressure (*p*/*p*_0_ < 0.01) confirms the microporosity. The Brunauer–Emmett–Teller (BET) surface areas of Zr-TBAPy, Zr-TCPE and Zr-TCPP are 1203, 842 and 265 m^2^ g^−1^, respectively. According to the Horvath–Kawazoe method, the pore volumes were calculated as 0.413, 0.264, and 0.0733 cm^3^ g^−1^, and the pore widths are centred at around 0.61, 0.50, and 0.64 nm, for Zr-TBAPy, Zr-TCPE and Zr-TCPP, respectively.

**Fig. 5:**
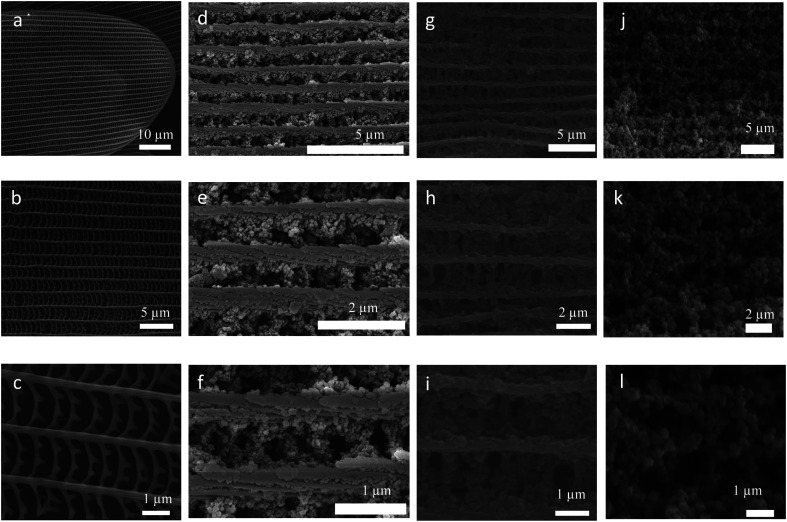
SEM image of (a–c) original butterfly wings, (d–f) Zr-TBAPy/B, (g–i) Zr-TCPE/B, (j–l) Zr-TCPP/B.

As photonic crystal, butterfly wing has a three-dimensional complex network and enable control and manipulation of light for photonic and optical applications.^52–56^ The present gels were employed to grow Zr-MOF nanoparticles on the surface of butterfly wing (*Euploea mulciber*) to obtain composite materials Zr-MOFs/B. It was expected to further disperse the MOF nanoparticles and promote the accessibility of guest molecules (thus for improved luminescence performance). The procedure for the synthesis of butterfly wing covered by Zr-TBAPy (denoted as Zr-TBAPy/B) is illustrated in Fig. S8.† The butterfly wing was first undergoing surface amination by (3-aminopropyl)triethoxysilane (APTES) to anchor zirconium ions. Subsequently, the modified butterfly wing was immersed in the Zr-TBAPy gel precursor solution. After ultrasonication for 20 min and heating at 100 °C overnight Zr-TBAPy/B was obtained. SEM shows that the size between two adjacent main ridges, which are composed of chitins,^57^ is approximately 1.2 μm for the original butterfly wing (Fig. 5). After the original butterfly wing was covered with Zr-TBAPy, the size decreased by approximately 600 nm between two adjacent ridges, demonstrating that Zr-TBAPy nanoparticles were successfully attached onto the surface of the ridges. Zr-TBAPy nanoparticles in Zr-TBAPy/B have narrower size distribution in the range of 120 to 140 nm than those in the xerogel (Fig. 5 and S9†). PXRD shows that NU-901 nanoparticles were grown oriented along the ridges (Fig. S3 and S10†). NU-901 nanoparticles in Zr-TBAPy/B were calculated to be 50 nm according to the Scherrer equation. Similarly Zr-TCPE/B and Zr-TCPP/B were prepared. For Zr-TCPP/B, PCN-224 nanoparticles were located on the surface of butterfly wing in the range of 50 to 100 nm (SEM) (Fig. 5, S11 and S12†). FT-IR spectra of Zr-TBAPy/B, Zr-TCPP/B, Zr-TCPE/B and the original butterfly wing were collected with attenuated total reflection (ATR) accessory (Fig. S13 and S14†). In this mode, the absorptions of covered butterfly wing could not be revealed. Similar to those of the xerogels, the stretching modes of carboxylate in Zr-TBAPy/B, Zr-TCPP/B and Zr-TCPE/B were observed at around 1655 cm^−1^ and 1410 cm^−1^, and the absorptions at around 720 and 650 cm^−1^ are assigned to Zr–O bonds.

**Fig. 6:**
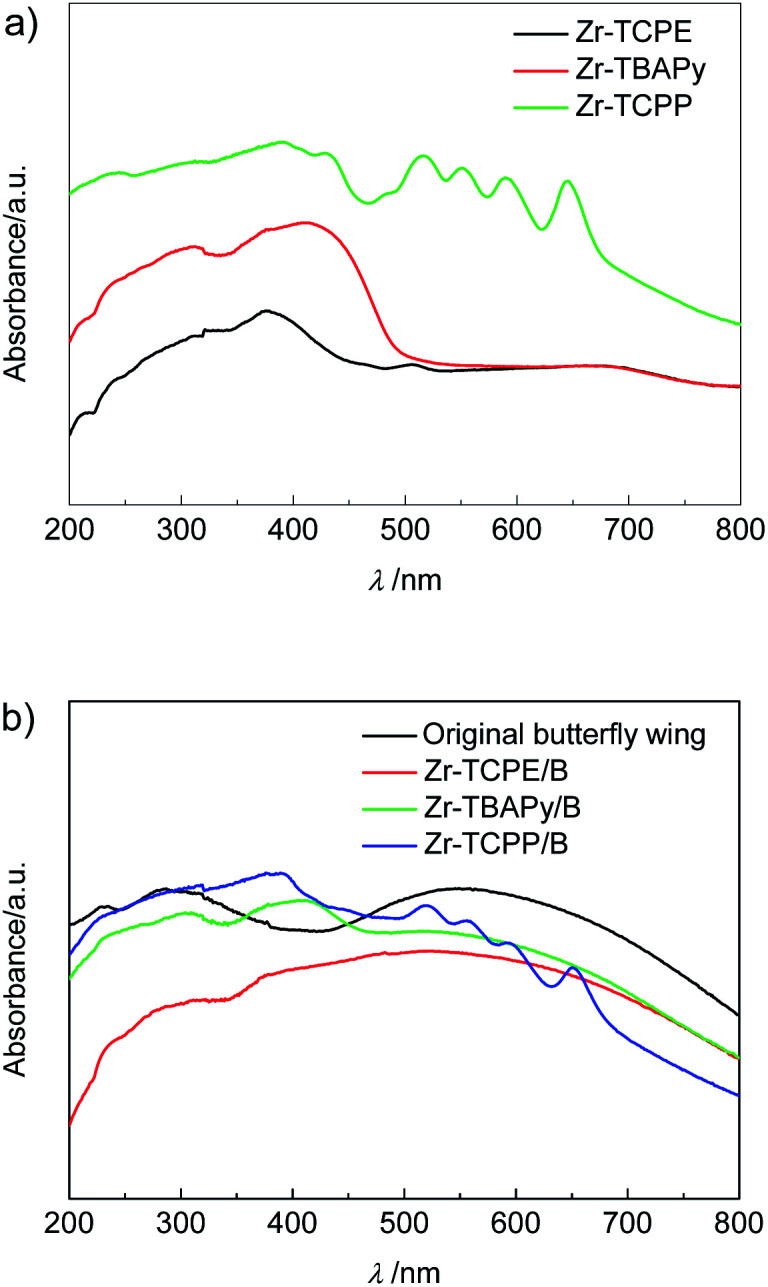
Diffuse reflectance spectra of (a) Zr-TCPE, Zr-TBAPy and Zr-TCPP xerogels, and (b) Zr-TCPE/B, Zr-TBAPy/B, Zr-TCPP/B and original butterfly wing.

Diffuse reflectance spectra of Zr-TBAPy, Zr-TCPE, Zr-TCPP xerogels and Zr-TBAPy/B, Zr-TCPE/B, Zr-TCPP/B are displayed in Fig. 6. Zr-TBAPy shows two absorption peaks beginning at 310 nm and maximizing at 412 nm with calculated *E*_g_ of 2.50 eV (Fig. S15†), which is consistent with the reported NU-901 MOF.^41,58^ The absorption maximum of Zr-TCPE is located at 375 nm, with calculated *E*_g_ of 2.69 eV. Zr-TCPP shows strong adsorption in the range of 300–800 nm with calculated *E*_g_ of 1.80 eV, which is consistent with the reported PCN-224(Zn).^59^ Zr-TBAPy/B, Zr-TCPE/B and Zr-TCPP/B exhibit absorption at about 590 nm in the visible light region which is derived from the butterfly wing template, and the redundant absorption is consistent with Zr-TBAPy, Zr-TCPE and Zr-TCPP. According to the diffuse reflectance spectra *E*_g_ is calculated to be 1.30 eV, 1.33 eV and 1.27 eV for Zr-TBAPy/B, Zr-TCPE/B and Zr-TCPP/B, respectively. The results show that *E*_g_ is significantly reduced in the presence of butterfly wing template.

Solid-state luminescent property of Zr-TBAPy and Zr-TCPE xerogels was investigated at room temperature (Fig. S16†). Excitation spectra show broad absorption band around 465 and 395 nm for Zr-TBAPy and Zr-TCPE xerogels, respectively. Emission spectra of Zr-TBAPy and Zr-TCPE exhibited strong emission at 520 and 498 nm, respectively. For Zr-TBAPy, the luminescence is centred on the highly conjugated pyrene core ligand of the framework, and may be assigned to pure intra-ligand emission, ligand to metal or metal to ligand charge transfer.^60,61^ For Zr-TCPE, the rigid framework efficiently restrict the intramolecular rotations, vibrations, and motions and avoid the self-quenching of tetraphenyl ethylene, thus resulting in the efficient aggregation-induced emission.^61,62^

**Fig. 7:**
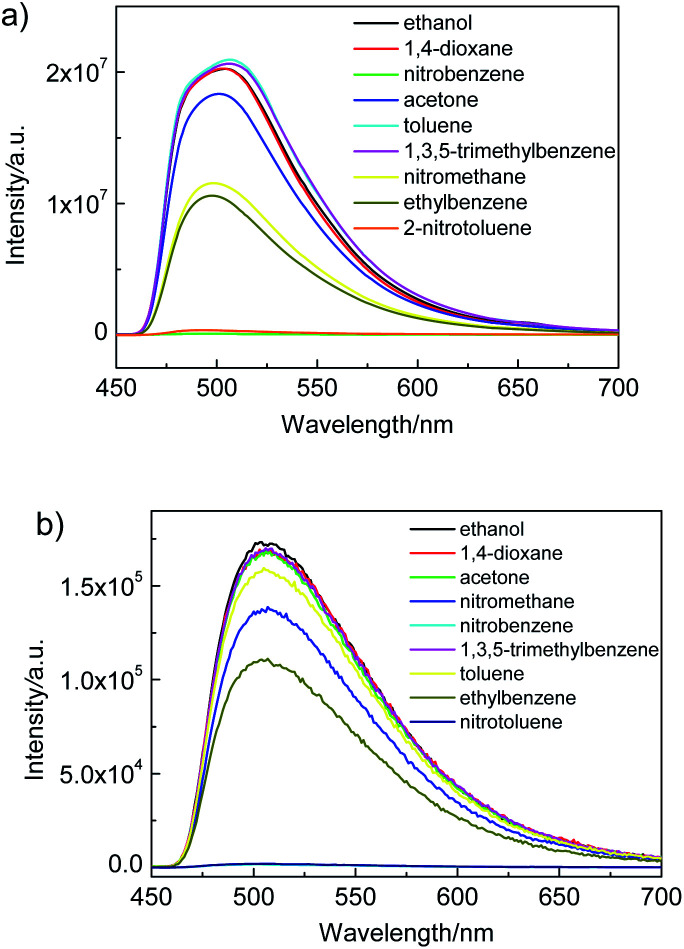
Luminescence response of (a) Zr-TBAPy (*λ*_ex_ = 465 nm) and (b) Zr-TCPE (*λ*_ex_ = 395 nm) in ethanol following exposure to various organic solvents.

To illustrate the ability of luminescence responses to various chemical species, the as-synthesized Zr-TBAPy and Zr-TCPE xerogels were dispersed in ethanol and exposed to a series of organic solvents including 1,4-dioxane, acetone, nitrobenzene, 2-nitrotoluene, nitromethane, toluene, ethylbenzene and 1,3,5-trimethylbenzene. As shown in Fig. 7, the intensity of the emission from Zr-TCPE and Zr-TBAPy xerogel depended heavily on the solvent that the sample was exposed to. Among the chemicals tested, complete quenching (quenching efficiency > 99%) was observed only for the nitroaromatic solvents, nitrobenzene and 2-nitrotoluene. These results indicate that Zr-TCPE and Zr-TBAPy xerogels may be used for the selective detection of nitrobenzene and 2-nitrotoluene in solution. In contrast, considerable enhancement effect was observed for electron donating analytes, and toluene and 1,3,5-trimethylbenzene induced large emission enhancement for Zr-TBAPy. The electron-donating analytes permits energy transfer to move the absorbed excitation energy from the analyte molecules onto xerogel, enhancing the material's emission. In the case of the nitro-functionalized analytes, reductive energy transfer from the xerogel to the strongly reducing nitro-analyte is an established mechanism by which these structures cause quenching in luminescent materials.

**Fig. 8:**
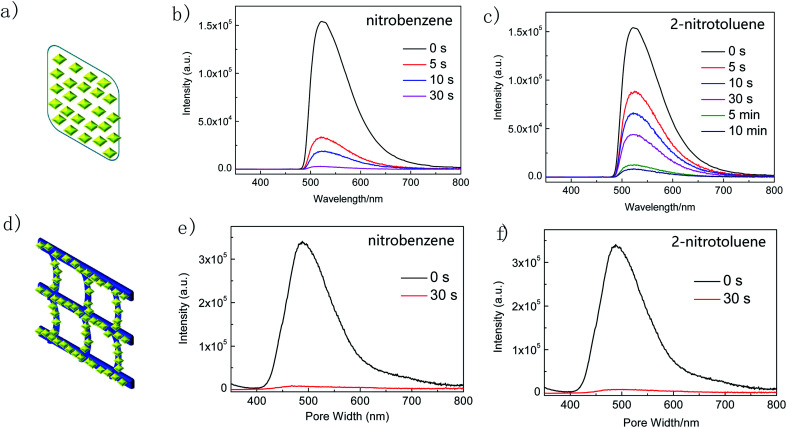
(a) Schematic representation of the film device of Zr-TBAPy, luminescence emission spectra of Zr-TBAPy xerogel after exposure to (b) nitrobenzene and (c) 2-nitrotoluene at different time intervals at room temperature, (d) schematic representation of Zr-TBAPy/B, and luminescence emission spectra of Zr-TBAPy/B after exposure to (e) nitrobenzene and (f) 2-nitrotoluene at different time intervals at room temperature (*λ*_ex_ = 465 nm).

Inspired by the excellent nitroaromatic sensing performance of Zr-TBAPy and Zr-TCPE xerogels in solution we investigated the nitroaromatic detecting ability in the vapour state. First we implemented the real-time solid-gas monitoring of nitrobenzene vapour by using a film device (Fig. 8). The film device was made easily through drop-casting a xerogel suspension in ethanol onto the quartz slide (as support), which was dried naturally. As the vapour concentration increased, the emission of Zr-TBAPy xerogel at 520 nm decreased significantly (Fig. S17†), indicating a typical phenomenon of energy transfer *via* intermolecular interaction. The luminescence intensity at 520 nm is basically linearly dependent upon the concentration of the nitrobenzene vapour at least within the concentration range studied (0–7 μmol L^−1^). The detection limit was calculated to be 0.1559 μmol L^−1^ based on the Stern–Volmer plots between (*I*_0_ − *I*)/*I* and analyte concentration according to the equation LOD = 3*σ*/*k*_sv_ (where *σ* is the standard deviation, 9.828 × 10^−3^, and *k*_sv_ is the slope of (*I*_0_ − *I*)/*I vs.* analyte concentration, 0.18908 L μmol^−1^).

The time-dependent luminescence emission intensity of Zr-TBAPy xerogel decreases continuously upon exposure to nitrobenzene and 2-nitrotoluene saturated vapour. The quenching efficiency (%) was estimated using the formula (*I*_0_ − *I*)/*I*_0_ × 100%, where *I*_0_ and *I* are the maximum luminescent intensity of xerogel before and after exposure to nitrobenzene and 2-nitrotoluene, respectively. Zr-TBAPy xerogel has 98% luminescence decrement after 30 s for nitrobenzene and 71% luminescence decrement after 30 s for 2-nitrotoluene. The emission intensity of complex Zr-TCPE xerogel also decreased continuously upon exposure to nitrobenzene saturated vapour (Fig. S18†). Nitrobenzene quenches the emission of Zr-TCPE by as much as 75% after 10 min and 72% after 10 min for 2-nitrotoluene. For the composite Zr-TBAPy/B, the quenching efficiency of nitrobenzene was 98% after 30 s which is equal to the Zr-TBAPy xerogel film, while the quenching efficiency for 2-nitrotoluene was 97% after 30 s which is higher than the Zr-TBAPy xerogel film (Fig. 8).

The short response time of Zr-TBAPy is comparable to some outstanding MOFs reported for the nitrobenzene vapour detection.^63,64^ The microporous diamond-shaped channels of NU-901 nanoparticles with the pore size of 2.9 nm × 1.2 nm is large enough to incorporate the target organic solvent molecules.^65^ For the film device, the sensing time is longer for 2-nitrotoluene than for nitrobenzene, probably attributed to the larger size of 2-nitrotoluene and its slower mass transfer in the porosity of Zr-TBAPy. When the nanoparticles are deposited on the butterfly wing template, the MOF nanoparticles are further dispersed by the network, the mass transfer may be greatly improved, and the sensing time is further shortened for 2-nitrotoluene.

## Conclusions

In summary, a series of Zr-carboxylate gels, namely Zr-TBAPy, Zr-TCPE, Zr-TCPP have been synthesized from pyrene, porphyrin and tetraphenyl ethylene-derived rigid tetracarboxylate linker. The gels are aggregated from MOF nanoparticles. PXRD shows that Zr-TBAPy gel consists of NU-901 nanoparticles, and Zr-TCPP gel consists of PCN-224 nanoparticles. The BET surface areas of Zr-TBAPy, Zr-TCPE and Zr-TCPP xerogels are 1203, 842 and 265 m^2^ g^−1^, respectively. In addition, the metal–organic gels were anchored on the surface of the pre-modified butterfly wing template, resulting in the formation of MOF gel films on the butterfly wing. Zr-TBAPy and Zr-TCPE gels are luminescent for solution-phase sensing and vapour-phase sensing of volatile organic compounds. Remarkably, Zr-TBAPy and Zr-TCPE exhibit significant luminescence quenching effect for electron-deficient analytes. Especially, a fast and effective vapour-sensing of nitrobenzene and 2-nitrotoluene have been achieved. Zr-TBAPy xerogel film has 98% luminescence decrement after 30 s exposure to nitrobenzene vapour. Zr-TBAPy/B shows >97% luminescence decrement after 30 s exposure to either nitrobenzene or 2-nitrotoluene vapour due to the high porosity and good dispersion of luminescent MOF nanoparticles. Such metal–organic gelation represents a promising approach to fabricate functional nanomaterials for efficient and selective sensing.

## Experimental

### Materials and methods

All chemicals were purchased from commercial sources and were used without further purification unless otherwise stated. Tetrakis(4-carboxyphenyl)ethylene (H_4_TCPE) was synthesized from tetraphenylethylene,^66–68^ tetrakis(4-carboxylphenyl)pyrene (H_4_TBAPy) was synthesized from 1,3,6,8-tetrabromopyrene^43^ and tetrakis(4-carboxyphenyl)porphyrin (H_4_TCPP)^69^ were synthesized according to the literature methods. Scanning electron microscopy (SEM) was performed using an ultra-high resolution SU8010 FE-SEM (Hitachi Instruments, working voltage is 5 kV, working current is 5 mA). Samples were treated *via* Au sputtering before observation. Powder X-ray diffraction (PXRD) was recorded on a Rigaku Smart Lab diffractometer at a rate of 5° min^−1^ (Bragg–Brentano geometry, Cu-Kα1 radiation, *λ* = 1.54056 Å, working voltage is 40 kV, working current is 15 mA). Infrared spectra were recorded using a Fourier transform infrared spectrometer in combination with an attenuated total reflection (ATR) unit from PerkinElmer Frontier. The sample was intimately pressed on the surface of the speculum of diamond crystal by a high-pressure clamp. The spectrum was collected after 32 scans in the range of 4000–650 cm^−1^. The UV-Vis absorption spectra of the MOF/B materials were recorded on a Shimadzu 2450 UV-Vis spectrometer under the diffuse-reflection model using an integrating sphere coated with BaSO_4_. The photoluminescence excitation and emission spectra were recorded using an FLS 920-combined time resolved and steady state fluorescence spectrometer (Edinburgh) equipped with photomultiplier tube operating at 400 V and a 150 W xenon was used as the excitation source. The temperature-dependent emission spectra were also collected on the same instrument with a temperature controller. Nitrogen adsorption–desorption isotherms for the materials were obtained at 77 K on a Micromeritics 3Flex system. The samples were evacuated at 393 K for 12 h prior to the measurements.

### Synthesis of Zr-TBAPy gel

ZrOCl_2_·8H_2_O (32.3 mg, 0.1 mmol) was dissolved in 1 mL of DMF. Benzoic acid (61.0 mg, 0.5 mmol), 1,3,6,8-tetrakis(*p*-benzoic acid)pyrene (34.1 mg, 0.05 mmol) and 25 μL of HCl (37 wt%) were added, after which the mixture was placed in an ultrasound bath for 15 min. The resulting mixture was then allowed to stand at 100 °C for gelation in a capped vial. A yellow gel was obtained after 1 h at 100 °C. After gelation, the wet gel was aged for 24 h at 100 °C. The gel was subsequently washed with DMF. DMF was replaced every day by fresh solvent and solvent exchange was finished after 3 d. Then the gel was washed with anhydrous EtOH for 3 d in similar way. The resulting xerogel was dried in vacuum (52.8 mg, yield 80%).

### Synthesis of Zr-TCPE gel

ZrOCl_2_·8H_2_O (32.3 mg, 0.1 mmol) was dissolved in 1 mL of DMF. Benzoic acid (61 mg, 0.5 mmol), H_4_TCPE (25.4 mg, 0.05 mmol) and 25 μL of HCl (37 wt%) were added, after which the mixture was placed in an ultrasound bath for 10 min. The resulting homogeneous solution was then allowed to stand at 100 °C for gelation in a capped vial. A yellow gel was obtained after 2 h at 100 °C. After gelation, the wet gel was aged for 24 h at 100 °C. The gel was subsequently washed with DMF. DMF was replaced every day by fresh solvent and solvent exchange was finished after 3 d. Then the gel was washed with anhydrous EtOH for 3 d in similar way. The resulting xerogel was dried in vacuum (48.0 mg, yield 83%).

### Synthesis of Zr-TCPP gel

ZrOCl_2_·8H_2_O (32.3 mg, 0.1 mmol) was dissolved in 1 mL of DMF. H_4_TCPP (39.5 mg, 0.05 mmol), 25 μL of HCl (37 wt%) and benzoic acid (61 mg, 0.5 mmol) were added, after which the mixture was placed in an ultrasound bath for 15 min. The resulting green mixture was then allowed to stand at 100 °C for gelation in a capped vial. A black gel was obtained after ∼2 h at 100 °C. After gelation, the wet gel was aged for 24 h at 100 °C. The gel was subsequently washed with DMF at room temperature. DMF was replaced every day by fresh solvent and solvent exchange continued for 3 d. Then the gel was washed with anhydrous EtOH for 3 d in similar way. The resulting xerogel was dried in vacuum (50.3 mg, yield 70%).

### Synthesis of Zr-TBAPy/B

Fresh butterfly wings were pretreated by a softening process in the steam and afterwards air-dried. To fabricate MOFs/B, butterfly wings were first washed with dilute 10 vol% nitric acid, washed in deionized water and dried in air. Then we used volume fraction 25% APTES diluted with absolute ethyl alcohol to activate the butterfly wings at 80 °C for 5 h, and washed with ethanol. The surface-amination-butterfly wings were then soaked in absolute ethyl alcohol saturated with ZrCl_4_ for 5 h at 80 °C, rinsed in absolute ethyl alcohol, and air-dried.

ZrOCl_2_·8H_2_O (162 mg, 0.501 mmol) was dissolved in 9 mL DMF. Benzoic acid (214 mg, 1.75 mmol), 1,3,6,8-tetrakis(*p*-benzoic acid)pyrene (300 mg, 0.438 mmol) and 150 μL of HCl (37 wt%) were added, after which the mixture was placed in an ultrasound bath for 15 min. The modified butterfly wing was carefully immersed into the resultant yellow mixture. The resulting mixture was then allowed to stand at 100 °C for gelation in a capped vial. A yellow gel was obtained after ∼1 h at 100 °C. After gelation, the system was aged for 24 h at 100 °C. The resulting Zr-TBAPy/B material was subsequently washed with DMF at room temperature. DMF was replaced every 12 h by fresh solvent and solvent exchange was finished after 3 times. Then the material was washed with anhydrous EtOH for 3 times in a similar way, and dried in vacuum.

Zr-TCPE/B and Zr-TCPP/B were synthesized from ZrOCl_2_·8H_2_O (323 mg, 1 mmol), H_4_TCPE (254 mg, 0.5 mmol) or H_4_TCPP (395 mg, 0.5 mmol), HCl (37 wt%, 250 μL) and benzoic acid (610 mg, 5 mmol) in 10 mL of DMF under analogous reaction procedures.

### Liquid-phase luminescence sensing measurements

The luminescence properties of Zr-xerogel dispersed in ethanol were investigated following exposure to various solvents. The ethanol dispersion was prepared by sonicating 5 mg of xerogel in 3 mL of ethanol for 30 min to create a stable dispersion. Following sonication, the emission curve was collected under 406 nm and 437 nm excitation for Zr-TCPE and Zr-TBAPy, respectively. 0.5 mL of the test solvent was then added, and the emission curve was recollected. The test solvents included ethanol, 1,4-dioxane, acetone, nitrobenzene, 2-nitrotoluene, toluene, ethylbenzene, nitromethane and 1,3,5-trimethylbenzene. All samples were well mixed with a pipet for 30 seconds before the emission curve was collected.

### Vapour-phase luminescence sensing measurements

First, 1 mL of each analyte was placed in small glass vials (20 mL) for several days to ensure that the equilibrium vapour pressure of each analyte was reached. 5 mg of xerogel in 0.5 mL ethanol were sonicated for 30 min then the dispersion was drop-casted onto the quartz slide, then was dried out naturally. For Zr-TBAPy/B, a piece of sample was directly attached on the quartz slide. The initial emission spectrum of solid sample was measured before it was exposed to vapours. Then, the sample slide was exposed to the vapour of analyte in a vial. After the specified exposure time, the slide was taken out from the vial and mounted into the sample holder of the luminescence spectrophotometer. The sample's luminescence emission spectrum was recorded immediately.

## Conflicts of interest

There are no conflicts to declare.

## Supplementary Material

RA-010-D0RA09035B-s001
