# Supplementary material for: Zirconium-based metal–organic framework gels for selective luminescence sensing
Source: RSC Adv. 2020 Dec 21;10(73):44912–9. doi: 10.1039/d0ra09035b (PMC9058640; doi:10.1039/d0ra09035b)
Supplement: RA-010-D0RA09035B-s001 [file RA-010-D0RA09035B-s001.pdf]

Electronic Supplementary Material (ESI) for RSC Advances.
This journal is © The Royal Society of Chemistry 2020

Electronic Supplementary Information

Zirconium-based metal-organic framework gels for selective luminescent sensing

Shujian Sun, Caifeng Wei, Yali Xiao, Guangqin Li and Jianyong Zhang*

Sun Yat-Sen University, MOE Laboratory of Polymeric Composite and Functional Materials, School of Materials Science and Engineering, School of Chemical Engineering and Technology, School of Chemistry, Guangzhou 510275, China. E-mail: zhjyong@mail.sysu.edu.cn.

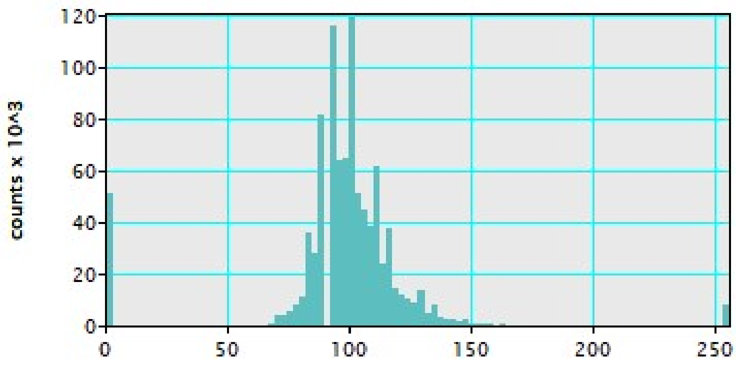

Fig. S1 Statistical particle size distribution of Zr-TBAPy xerogel.

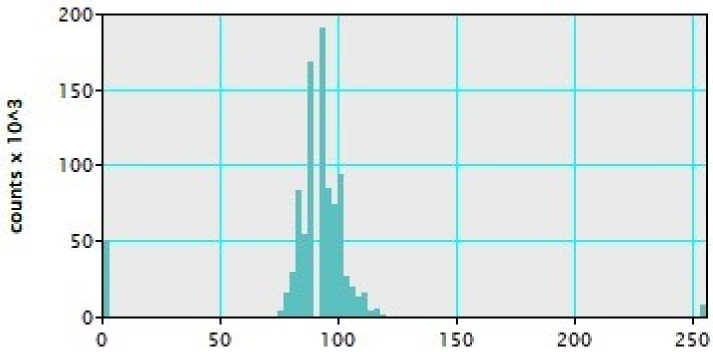

Fig. S2 Statistical particle size distribution of Zr-TCPE xerogel.

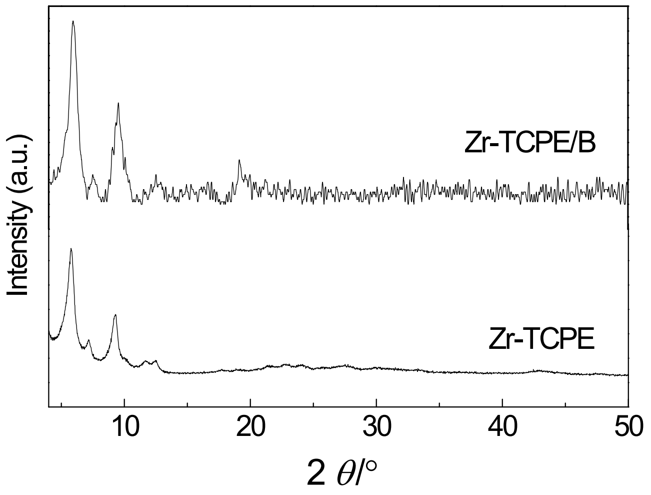

Fig. S3 PXRD patterns of Zr-TCPE xerogel and Zr-TCPE/B.

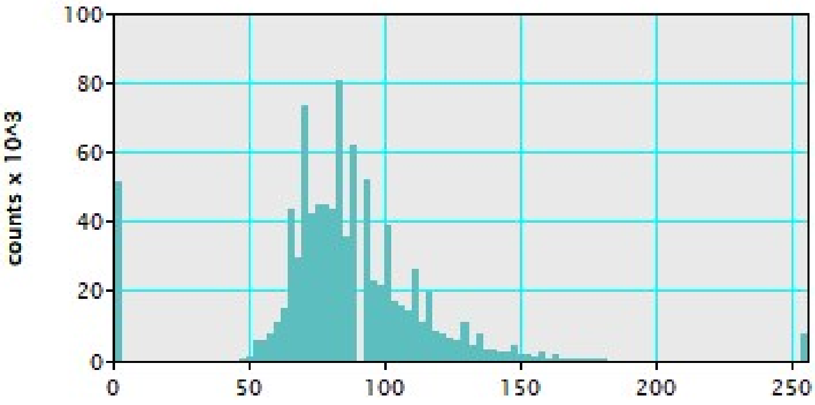

Fig. S4 Statistical particle size distribution of Zr-TCPP xerogel.

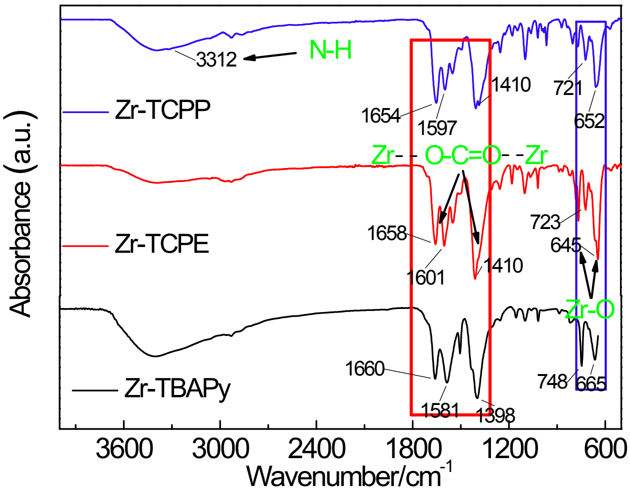

Fig. S5 FT-IR spectra of Zr-TBAPy, Zr-TCPE and Zr-TCPP xerogels.

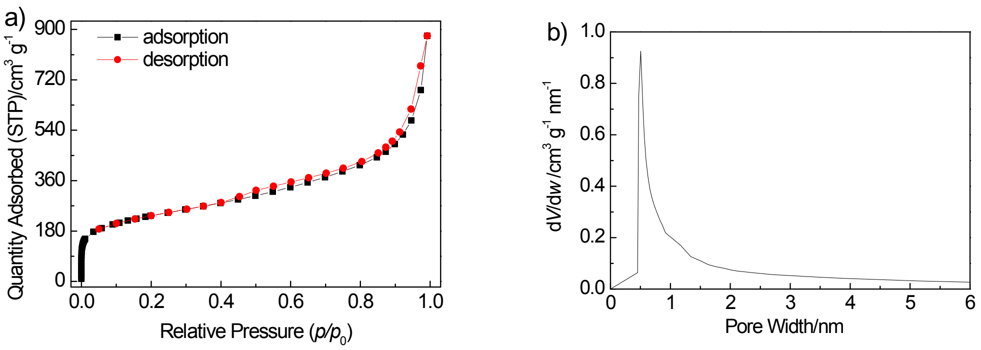

Fig. S6 a) N_2 adsorption-desorption isotherms and b) Horvath-Kawazoe micropore analysis of Zr-TCPE xerogel.

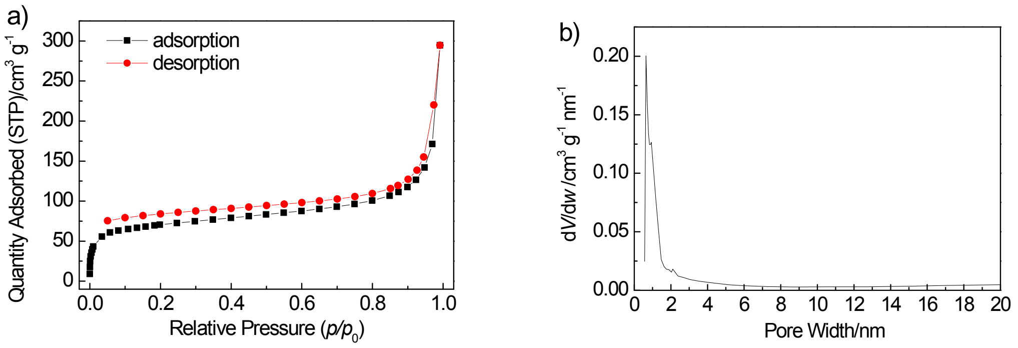

Fig. S7 a) N_2 adsorption-desorption isotherms and b) Horvath-Kawazoe micropore analysis of Zr-TCPP xerogel.

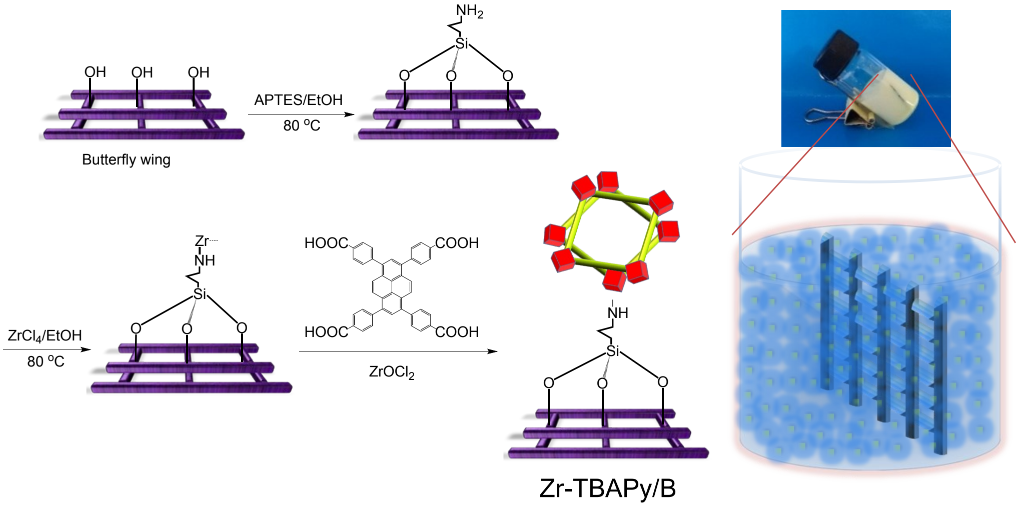

Fig. S8 Schematic diagram to illustration the synthetic route of Zr-TBAPy/B.

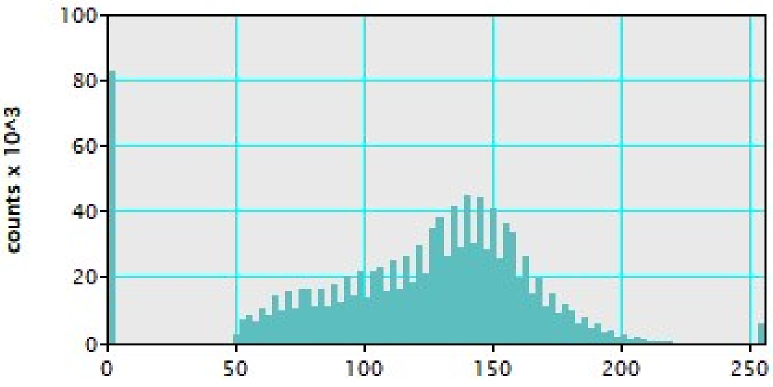

Fig. S9 Statistical particle size distribution of Zr-TBAPy/B.

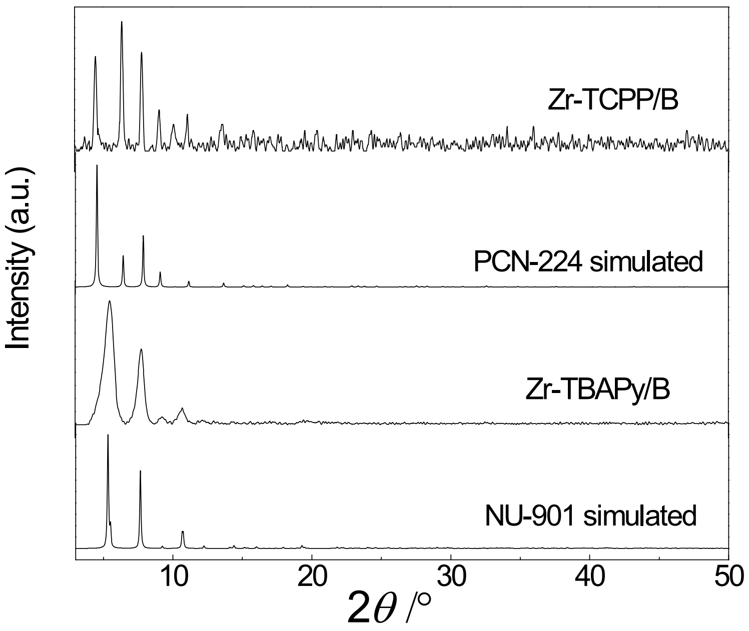

Fig. S10 PXRD patterns of Zr-TBAPy/B and Zr-TCPP/B.

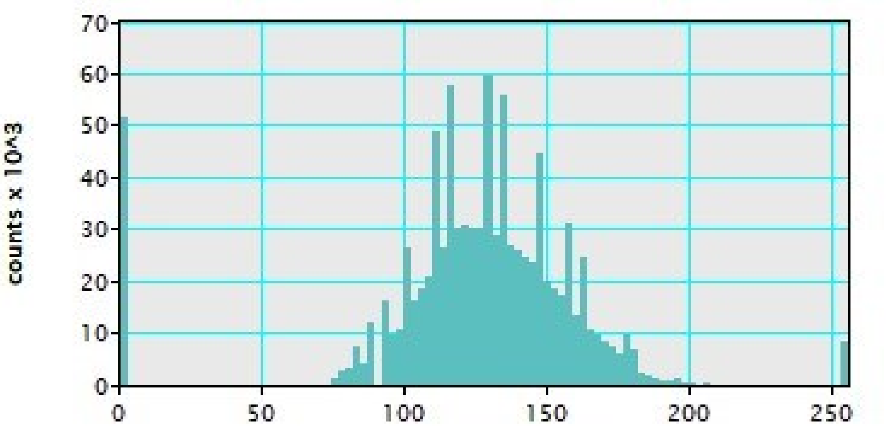

Fig. S11 Statistical particle size distribution of Zr-TCPE/B.

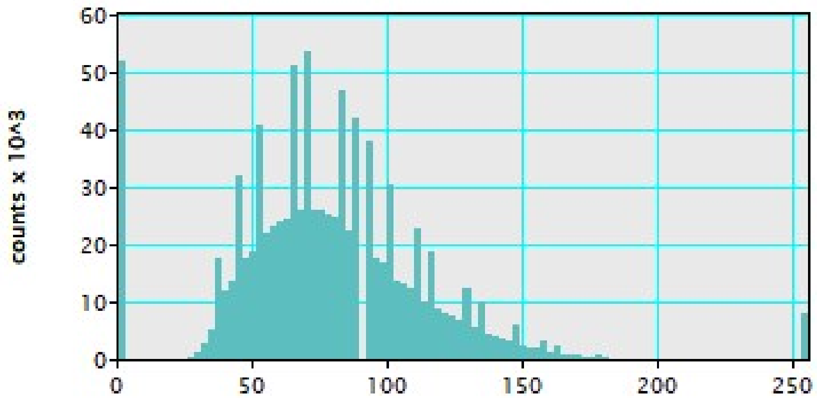

Fig. S12 Statistical particle size distribution of Zr-TCPP/B.

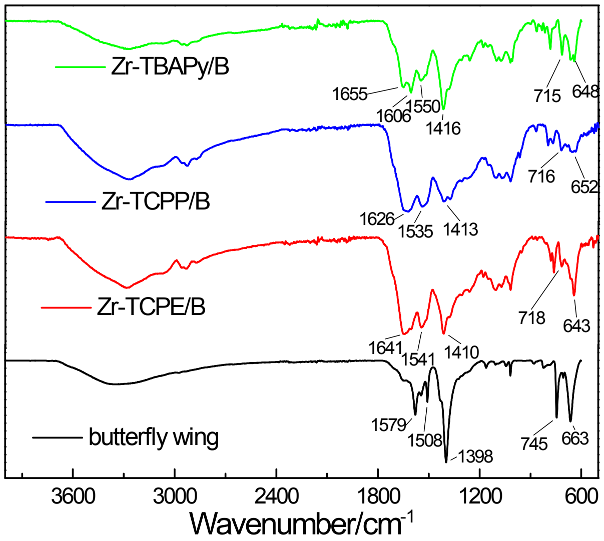

Fig. S13 FT-IR spectra of Zr-TBAPy/B, Zr-TCPP/B, Zr-TCPE/B and the original butterfly wing.

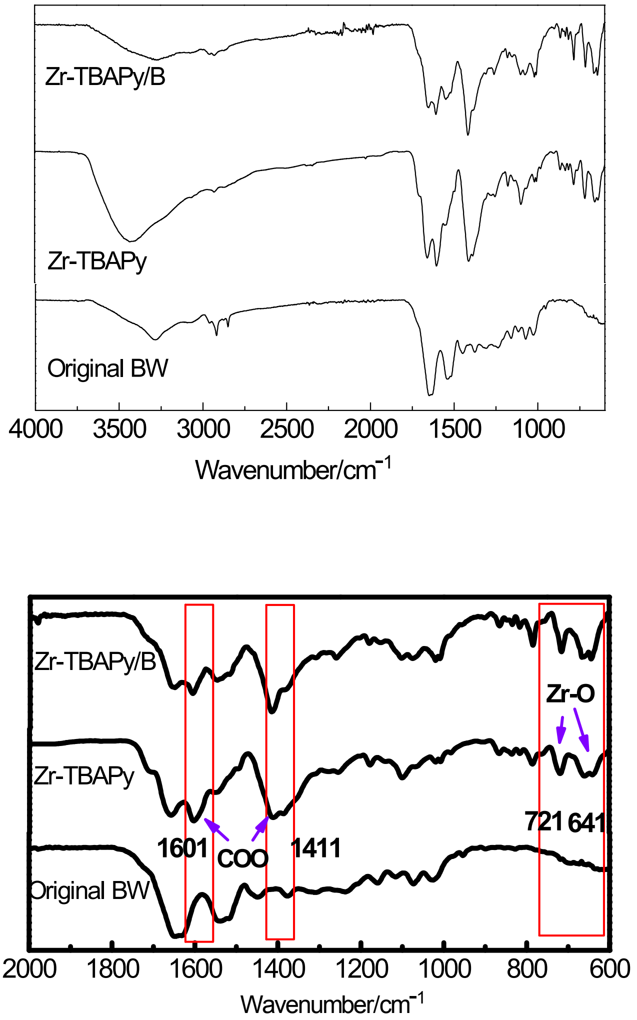

Fig. S14 FT-IR spectra of Zr-TBAPy and Zr-TBAPy/B.

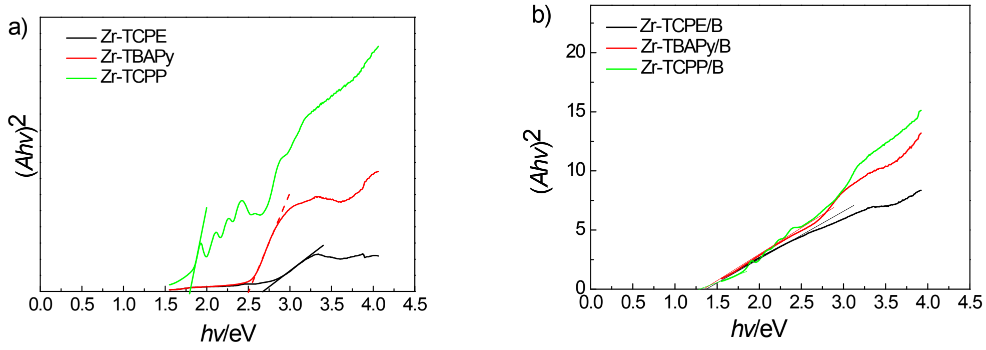

Fig. S15 Energy gap calculation of a) Zr-TCPE, Zr-TBAPy and Zr-TCPP xerogels, and b) Zr-TCPE/B, Zr-TBAPy/B and Zr-TCPP /B.

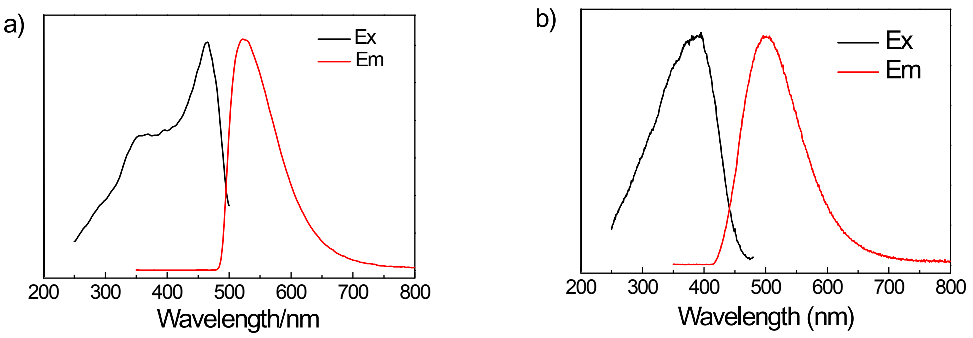

Fig. S16 Luminescence excitation and emission spectra of a) Zr-TBAPy xerogel (λ_{em} = 520 nm, λ_{ex} = 465 nm) and b) Zr-TCPE xerogel (λ_{em} = 498 nm, λ_{ex} = 395 nm) at room temperature.

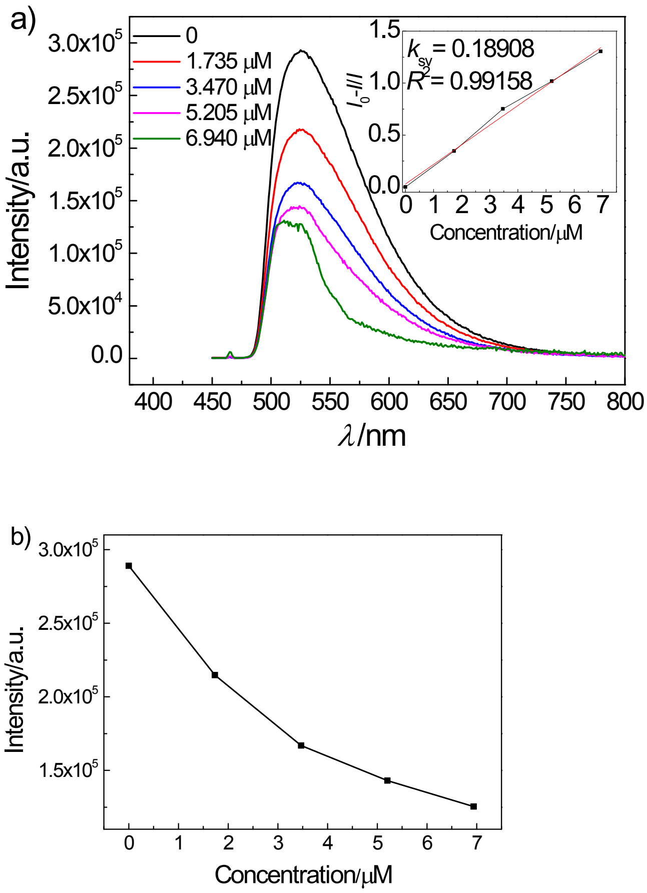

Fig. S17 a) Concentration-dependent emission spectra at room temperature (λ_{ex} = 465 nm) after the Zr-TBAPy xerogel film device was exposed in nitrobenzene vapour for 5 s. The inset shows the plot of the fluorescence intensity $(I_0 - I)/I$ at 520 nm against *c*. b) the plot of the emission intensity at 520 nm against *c*.

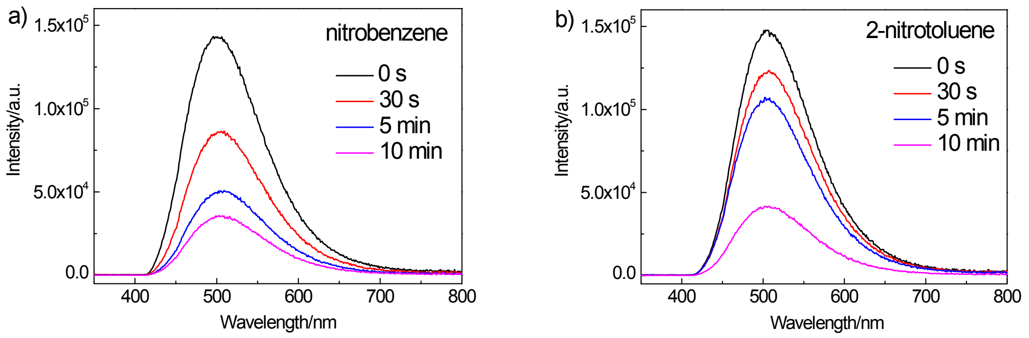

Fig. S18 Luminescence emission spectra of Zr-TCPE xerogel after exposure to a) nitrobenzene vapour and b) 2-nitrotoluene vapour at different time intervals at room temperature (λ_{ex} = 395 nm).
